# Positive Association of Leptin and Artery Calcification of Lower Extremity in Patients With Type 2 Diabetes Mellitus: A Pilot Study

**DOI:** 10.3389/fendo.2021.583575

**Published:** 2021-05-19

**Authors:** SanBao Chai, Yao Chen, SiXu Xin, Ning Yuan, YuFang Liu, JianBin Sun, XiangYu Meng, YongFen Qi

**Affiliations:** ^1^ Department of Endocrinology and Metabolism, Peking University International Hospital, Beijing, China; ^2^ Laboratory of Cardiovascular Bioactive Molecule, School of Basic Medical Sciences, Peking University, Beijing, China; ^3^ Key Laboratory of Molecular Cardiovascular Science, Ministry of Education, Peking University Health Science Center, Beijing, China; ^4^ Department of Pathogen Biology, School of Basic Medical Sciences, Peking University, Beijing, China; ^5^ The Central Laboratory, Peking University International Hospital, Beijing, China

**Keywords:** leptin, type 2 diabetes mellitus, VSMC, artery calcification, phenotypic switch

## Abstract

**Objective:**

We aimed to explore the role and possible mechanism of leptin in lower-extremity artery calcification in patients with type 2 diabetes mellitus (T2DM).

**Methods:**

We recruited 59 male patients with T2DM and 39 non-diabetic male participants. All participants underwent computed tomography scan of lower-extremity arteries. The calcification scores (CSs) were analyzed by standardized software. Plasma leptin level was determined by radioimmunoassay kits. Human vascular smooth muscle cells (VSMCs) calcification model was established by beta-glycerophosphate and calcium chlorideinduction. Calcium deposition and mineralization were measured by the o-cresolphthalein complexone method and Alizarin Red staining. The mRNA expression of bone morphogenic protein 2 (BMP2), runt-related transcription factor 2 (Runx2), osteocalcin (OCN) and osteopontin (OPN) was determined by quantitative RT-PCR. The protein levels of BMP2, Runx2, α-smooth muscle actin (α-SMA) and (p)-Akt was determined by Western-blot analysis, and α-SMA was also measured by immunofluorescence analysis.

**Results:**

Compared with controls, patients with T2DM showed higher median calcification score in lower-extremity artery [286.50 (IQR 83.41, 1082.00) *vs* 68.66 (3.41, 141.30), *p*<0.01]. Plasma leptin level was higher in patients with calcification score ≥300 than ≥100 (252.67 ± 98.57 *vs* 189.38 ± 44.19 pg/ml, *p*<0.05). Compared with calcification medium, intracellular calcium content was significantly increased in VSMCs treated by leptin (200, 400 and 800 ng/ml) combined with calcification medium [11.99 ± 3.63, 15.18 ± 4.55, and 24.14 ± 5.85 mg/ml, respectively, *vs* 7.27 ± 1.54 mg/ml, all *p*<0.01]. Compared with calcification medium, Alizarin Red staining showed calcium disposition was more obvious, and the mRNA level of BMP2, Runx2 and OCN was significantly increased, and immunofluorescence and Western blot analysis showed that the expression of α-SMA was downregulated in VSMCs treated by leptin (400 ng/ml) combined with calcification medium, respectively. Compared with calcification medium, the protein level of BMP2 and Runx2 was upregulated in VSMCs treated by leptin (400 ng/ml) combined with calcification medium. Moreover, blocking PI3K/Akt signaling pathway can decrease the protein expression of BMP2 and Runx2 in VSMCs treated by leptin (400 ng/ml) combined with calcification medium.

**Conclusions:**

Leptin promoted lower-extremity artery calcification of T2DM by upregulating the expression of BMP2 and Runx2, and regulating phenotypic switch of VSMCs *via* PI3K/Akt signaling pathway.

## Introduction

The global prevalence of diabetes in adults has increased in recent decades. The number of patients 20 to 79 years old with diabetes is predicted to increase to 642 million by 2040 (1). Arteriosclerotic calcification is increased in type 2 diabetes mellitus (T2DM), which impairing vascular compliance and function, thereby increasing the risk for heart attack, stroke, limb ischemia, and lower extremity amputation (2, 3). Arterial calcification has long been considered a passive process, but now it is known to be an active regulatory process of tissue biomineralization.

Recently, adipose tissue is recognized as a biologically active tissue. Adipose tissue is metabolically active and produces a multitude of adipokines, which may direct effects on the skeleton as well as on vascular structure and function (4). Previous reports indicated that abdominal obesity predicts coronary artery calcification presence and progression (5, 6). Clinical studies suggested that adipose tissue plays an important role in the formation of vascular calcification (VC) (7–9). Although the clinical effects of VC have been well documented, whether adipokines involved in the progression of VC is still unclear. Leptin, a 16-kDa non-glycosylated protein, is encoded by the *ob* gene and mainly secreted by adipocytes (10). The central function of leptin is to inhibit food intake and appetite, and the peripheral effects are mainly related to insulin resistance, inflammatory response, oxidative stress, atherosclerosis (11, 12). Plasma leptin level has a close relationship with the occurrence and development of obesity, metabolic syndrome and hypertension (13, 14). Previous studies implicated a link between high leptin concentrations and increased risk of cardiovascular as well as microvascular complications (14, 15). Vascular calcification can be divided into intimal calcification and media calcification in morphology, and media calcification is the most common type of vascular calcification in patients with T2DM. Oxidative stress, endothelial dysfunction, abnormal mineral metabolism and increased inflammatory factors are involved in the process of vascular calcification (16). Previous studies have found that adipokines play an important role in vascular calcification, and confirmed that leptin regulates the osteoblastic differentiation and calcification of vascular cells (17). Moreover, leptin is associated with insulin resistance and demonstrated as a significant independent predictor of coronary artery calcification (18).

The key link in the pathophysiological process of vascular calcification is the phenotypic transformation of smooth muscle cells into osteoblast like cells (19, 20). The differentiation of vascular smooth muscle cells (VSMCs) into osteoblast like cells mediates the process of bone matrix deposition in blood vessels.

Bone morphogenetic protein (BMP) is a member of the transforming growth factor superfamily and plays an important role in mammalian bone development. Among them, BMP2-Smad1/5/8 signaling pathway plays a vital role in ectopic ossification (21, 22). The Runt-related transcription factor (Runx2) is a key regulator of normal bone development, homeostasis and remodeling. During osteogenic differentiation, Runx2 activated by BMP2 regulate the expression of osteoblast markers such as osteocalcin (OCN) and osteopontin (OPN) (23, 24). The cytoskeletal α-smooth muscle actin (α-SMA) is the actin isoform that predominates within VSMCs, which is one of the most well-established hallmarks and used to mark the phenotypic changes of myocytes (25, 26).

However, there is few information whether leptin is involved in the development of lower-extremity arterial calcification in T2DM. In the present study, we investigated changes of leptin level in T2DM with lower-extremity arterial calcification and its possible mechanisms involved in the development of T2DM-related vascular calcification.

## Materials and methods

### Materials

Human VSMC CRL1999 were purchased from the American Type Culture Collection (ATCC, Manassas, VA, USA). Protease and phosphatase inhibitors were purchased from Roche Applied Science (Basel, Switzerland). Recombinant human leptin was obtained from R&D Systems (398-LP-01, R&D Systems, MN, USA). Anti-BMP2 antibody, anti-α-SMA antibody, and anti-glyceraldehyde-3-phosphate dehydrogenase (GAPDH) antibody were purchased from Abcam (Cambridge, United Kingdom). Anti-Akt antibody, Anti-p-Akt (Ser473) antibody, Anti-Runx2 antibody was purchased from Cell Signaling Technology Inc. (Beverly, MA, USA). LY294002 was obtained from Merck (Sigma-Aldrich, St. Louis, MO, USA). 4’,6-diamidino-2-phenylindole (DAPI), tetramethylrhodamine isothiocyanate (TRITC)-conjugated goat anti-rabbit, and horseradish peroxidase (HRP)-conjugated goat anti-rabbit secondary antibodies were purchased from Beijing Zhongshan Golden Bridge Biological Technology Company (Beijing, China).

### Study design

All participants gave their informed consent for inclusion before they participated in the study. The study was conducted in accordance with the ethical guidelines of the 1975 Declaration of Helsinki, and the protocol was approved by the Ethics Committee of Peking University International Hospital (No. 2016-025). Participants had the right to refuse to participate in or withdraw from the study at any time. From January 2017 to December 2017, we recruited male participants with T2DM and non-diabetic male participants. The inclusion criteria of the diabetes group were males with T2DM ≥ 18 years old receiving oral hypoglycemic agents with or without insulin. Exclusion criteria were previous bypass surgery or percutaneous angioplasty to lower-limb arteries, acute limb ischemia, moderate or severe renal disease, significant hepatic disease, cardiorespiratory disease, malignancy, connective tissue disease, smoking, drinking, uncontrolled hypertension, or acute illness.

### Calcification Scores of Lower-Extremity Arteries

All participants underwent CT scan of lower extremity arteries with helical acquisition at kVp=120, mAs=200, and field of view 350 to 380 mm. From the acquired raw data, the scan was reconstructed in 5-mm-thick slices. The average number of slices of lower-extremity arteries was approximately 250. The scores for vascular calcification included external iliac, internal iliac, and superficial femoral arteries; deep femoral artery; popliteal arteries and peroneal arteries. Calcification scores (CSs) were analyzed by using standardized calcium scoring software (Intelligent portal space, Intellispace Portal, Philips Healthcare) by investigators who were blinded to clinical data. On cross-sectional images of the lower extremities, the area of calcification in a cross-sectional area >1 mm^2^ and density >130 hounsfield units was identified and scored. The CSs for each segment of interest was determined and expressed as an Agatston score (27).

### Sample Collection

Blood samples were collected from all participants on an empty stomach for 8 hours. Ethylene diamine tetraacetic acid-plasma was isolated by centrifugation at 3,000 rpm for 15 min. Aprotinin (500 units/ml; Sigma Co., St. Louis, MO, USA) was added into plasma for leptin analysis and samples were stored at −80°C.

### Plasma Radioimmunoassay

Plasma leptin level was determined by using commercially available radioimmunoassay kits (DIAsource, Belgium). According to the manufacturer’s protocol, the radioactive precipitation was counted by using a γ-counter. The intra- and inter-assay coefficients of variation were < 10% and 15%.

### Measurement of Other Parameters

Blood pressure was measured in the supine position for all participants. Levels of fasting blood glucose (FBG), high-density lipoprotein cholesterol (HDL-C), low-density lipoprotein cholesterol (LDL-C), triglyceride (TG), total cholesterol (TC), and glycosylated hemoglobin A1_C_ (HbA1_C_) were measured by routine biochemistry analysis.

### Cell Culture of Human VSMCs

Human VSMC CRL1999 were purchased from the American Type Culture Collection (ATCC, Manassas, VA, USA) and cultured in Dulbecco’s modified Eagle’s medium supplemented with 15% fetal bovine serum (Gibco, Life Technologies), 100 U/ml penicillin and 100 μg/ml streptomycin in a humidified atmosphere containing 5% CO_2_. VSMCs at passages 7-10 were used for the experiments.

### *In Vitro* Calcification and Quantification of VSMCs

VSMCs were cultured with calcification medium containing calcium chloride (5 mmol/l), β-glycerophosphate (10 mmol/l) and leptin (200, 400 and 800 ng/ml) or calcification medium alone. The calcification medium was replaced every 3 days for a total of 7 days. VSMCs were decalcified for 6 hr at 4°C in 0.6 mol/l HCl and calcium levels were determined by colorimetry with the o-cresolphthalein complexone method (Calcium Kit, Biosino Bio-Technology and Science, Beijing). Ca^2+^ concentration (mg/ml) in samples was normalized over the sample protein concentration (mg/ml) as determined by the Lowry assay.

### Alizarin-Red Staining

Calcification of human VSMCs was induced as previously described (28) with minor modification. After removing the medium, VSMCs grown in 12-well plates were washed with phosphate-buffered saline (PBS), fixed with 4% formaldehyde, and stained with alizarin red for 5 min, then washed again with distilled water and observed by microscopy.

### Immunofluorescence Analysis

Human VSMCs were fixed with 4% formaldehyde for 10 min, washed 3 times in PBS, permeabilized with 0.5% Triton X-100 for 15 min at room temperature, then preincubated with normal goat serum for 30 min and incubated with anti-α-SMA antibody (1:200 in PBS) overnight at 4°C. Then, cells were washed 3 times and incubated with TRITC conjugated goat anti-rabbit (594 nm, 1:200) antibodies for 1 h. Nuclei were stained using DAPI (1:1000 diluted in PBS) for 5 min. Cells on coverslips were then mounted on slides and examined with fluorescence microscope (Nikon).

### Quantitative RT-PCR (qRT-PCR)

qRT-PCR was used to determine the effect of leptin on the expression of bone-related gene markers in VSMCs. Total RNA was isolated from cultured VSMCs by using TRIzol Reagent (Invitrogen, Carlsbad, CA, USA) according to the manufacturer’s instructions. Reverse transcription involved use of an RT-PCR kit (Takara Biotech). Levels of mRNA were quantified by using an ABI 7500HT Fast Real-Time PCR System (Applied Biosystems, Grand Island, NY, USA). GAPDH was an endogenous control. The 2^–ΔΔCt^ method was used to calculate relative expression level. See **Table 1** for specific primers.

**Table 1:** Primers and protocols for qRT-PCR.

Genes	Primer sequence
Human BMP2	Fwd: TCAAGCCAAACACAAACAGC
	Rev: GGAGCCACAATCCAGTCATT
Human Runx2	Fwd: CCGTCCATCCACTCTACCAC
	Rev: ATGAAATGCTTGGGAACTGC
Human OCN	Fwd: CTCACACTCCTCGCCCTATT
	Rev: CGCCTGGGTCTCTTCACTAC
Human OPN	Fwd: GCCGTGGGAAGGACAGTTAT
	Rev: GCTCATTGCTCTCATCATTGG
Human GAPDH	Fwd: CAGGAGGCATTGCTGATGAT
	Rev: GAAGGCTGGGGCTCATTT

BMP2, bone morphogenic protein 2; Runx2, runt-related transcription factor 2; OCN, osteocalcin; OPN, osteopontin; GAPDH, glyceraldehyde-3-phosphate dehydrogenase.

### Western Blot Analysis

Protein extraction from cultured human VSMCs was prepared in radioimmunoprecipitation assay buffer supplemented with protease inhibitor and phosphatase inhibitor. Total protein was subjected to electrophoresis on sodium dodecyl sulfatepolyacrylamide gel electrophoresis gels and electrically transferred to nitrocellulose membranes. Then, membranes were blocked in 5% nonfat milk for 1 hour and incubated overnight at 4°C with the following primary antibodies: anti-BMP2 (1:200 dilution), anti-Runx2 (1:200 dilution), anti-Akt (1:1000 dilution), anti-p-Akt (1:1000 dilution), anti-α-SMA (1:1000 dilution), and anti-GAPDH (1:2000 dilution). The membranes were then incubated with the HRP-conjugated goat antirabbit immunoglobulin G secondary antibody for 1 hour, followed by detection with ECL (Millipore).

### Statistical Analysis

SPSS 23 (SPSS, Chicago, IL, USA) was used for statistical analysis. Data are tested for normality by Shapiro-Wilk test and expressed as mean ± SD or median (interquartile range [IQR]). Statistical analysis included independent *t*-test, paired *t*-test for normal distribution and Mann-Whitney U test for non-normal distribution. *p*<0.05 was considered statistically significant.

## Results

### Increased Lower-Extremity Artery Calcification Score and Leptin Level in Patients With T2DM

We included 59 males patients with T2DM and 39 non-diabetic male participants. As compared with controls, patients with T2DM showed higher median calcification score in lower-extremity artery [286.50 (IQR 83.41, 1082.00) *vs* 68.66 (3.41, 141.30), *p*<0.01], and median plasma leptin level [183.40 (130.00, 248.80) *vs* 139.00 (82.36, 218.30) pg/ml, *p*<0.05] (**Table 2**).

**Table 2:** Baseline characteristics of patients with type 2 diabetes mellitus and control group.

parameter	T2DM group	control group	*p* value
N	59	39	–
Age*	55.81 ± 14.74	53.74 ± 16.05	0.51
Duration (year)^#^	7.00(2.00, 13.00)	–	–
BMI (kg/m^2^)^#^	26.10(23.90, 28.60)	25.00(24.10,26.10)	0.11
SBP (mmHg)^#^	135.00(123.00, 145.00)	131.00(120.00,141.00)	0.50
DBP (mmHg)^#^	80.00(75.00, 89.00)	80.00(70.00,89.00)	0.38
FBG (mmol/L)^#^	7.87(6.39, 9.56)	5.55(5.02, 6.24)	<0.01
HbAlc (%)^#^	7.60(6.80,9.40)	5.60(5.20,5.80)	<0.01
ALT (U/L)^#^	22.00(16.00, 30.00)	25.00(16.00, 33.00)	0.81
AST (U/L)^#^	20.00(16.00, 28.00)	23.00(20.00, 30.00)	0.06
Cholesterol (mmol/L)^#^	4.18(3.35, 4.77)	3.96(3.52, 4.79)	0.98
Triglyceride (mmol/L)^#^	1.28(0.90, 2.13)	1.36(1.10, 1.90)	0.63
HDL-C (mmol/L)^#^	0.89(0.78, 1.10)	0.96(0.83, 1.04)	0.26
LDL-C (mmol/L)^#^	2.54(1.94, 3.00)	2.42(2.01, 3.08)	0.97
Leptin (pg/ml)^#^	183.40(130.00, 248.80)	139.00(82.36, 218.30)	<0.05
calcification scores^#^	286.50(83.41, 1082.00)	68.66(3.41, 141.30)	<0.01

T2DM, type 2 diabetes mellitus; BMI, body mass index; SBP, systolic blood pressure; DBP, diastolic blood pressure; FBG, fasting blood glucose; HbA1c, glycated hemoglobin A1_C_; ALT, alanine aminotransferase; AST, aspartate aminotransferase; HDL-C, high-density lipoprotein cholesterol; LDL-C, low-density lipoprotein cholesterol.

*data expressed as mean ± SD and tested by independent t-test for the normal distribution.

^#^data expressed as median (p25, p75) and tested by Mann-Whitney U test for the non-normal distribution.

### Plasma Leptin Level Associated With Severity of Lower-Extremity Arterial Calcification in T2DM

**Figure 1:**
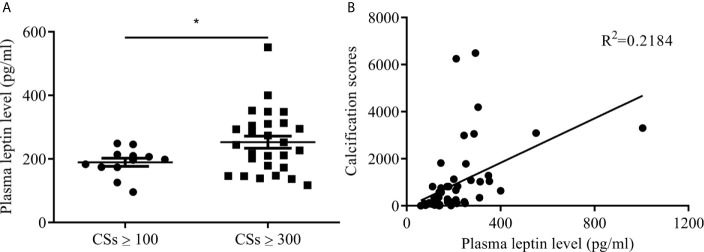
Plasma leptin levels in patients with T2DM and arterial calcification of lower extremity. **(A)** plasma leptin levels between calcification score ≥100 and ≥300. Data expressed as median (p25, p75), n=12 CSs ≥100, n=29 CSs ≥300, *p<0.05 *vs* CSs ≥100 **(B)** the correlation between plasma leptin levels and calcification scores. CSs, calcification scores; T2DM, type 2 diabetes mellitus.

Participants with T2DM were divided into two groups by lower-extremity arterial calcification scores: ≥100 and ≥300. Mean plasma leptin level was higher with calcification score ≥300 than ≥100 (252.67 ± 98.57 vs 189.38 ± 44.19 pg/ml, *p*<0.05) (**Figure 1A**). The correlation coefficient R^2^ was 0.2184 between plasma leptin and vascular calcification score (**Figure 1B**). These results indicated that plasma leptin level associated with severity of lower-extremity arterial calcification in T2DM.

### Leptin Promotes VSMCs Calcification *In Vitro*

**Figure 2:**
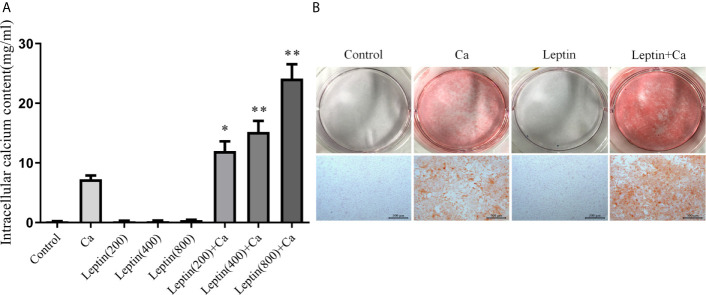
Leptin enhances calcification of VSMCs. **(A)** intracellular calcium content. Data are mean ± SD. n=6 independent experiments. *p<0.05, **p<0.01 *vs* Ca. **(B)** alizarin red staining (Scale bar=500 μm). VSMCs, vascular smooth muscle cells; Ca, calcification medium; Leptin (200), 200 ng/ml; Leptin (400), 400 ng/ml; Leptin (800), 800 ng/ml.

To investigate whether leptin could promote vascular calcification, human VSMCs were treated with various concentrations of leptin combined with calcification medium for 7 days. Calcium content was significantly increased with calcification medium combined with leptin 200, 400 and 800 ng/ml versus calcification medium alone [11.99 ± 3.63, 15.18 ± 4.55, and 24.14 ± 5.85 mg/ml, respectively, *vs* 7.27 ± 1.54 mg/ml, all *p*<0.01] (**Figure 2A**). After treated with leptin (400 ng/ml) and calcification medium for 7 days, Alizarin Red staining showed calcium disposition was more obvious than that in calcification medium alone (**Figure 2B**).

### Leptin Induced Phenotypic Switch of VSMCs *In Vitro*

**Figure 3:**
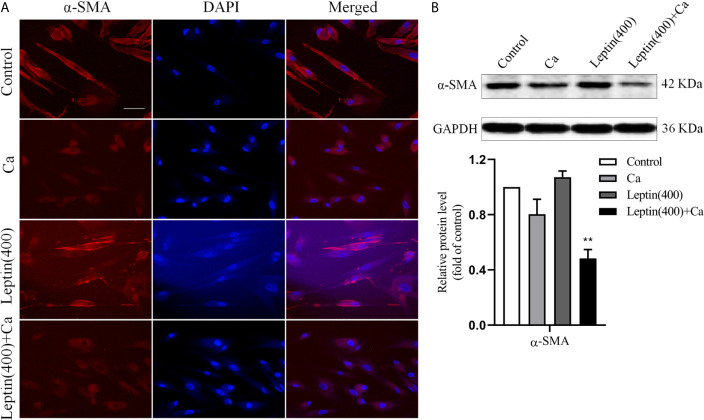
Leptin induced phenotypic switch of VSMCs *in vitro*. **(A)** representative immunofluorescence staining with antibodies for α-SMA in VSMCs (magnification: ×200) (Scale bar=25 μm). **(B)** Western blot assay for expression of α-SMA in VSMCs. Data are shown as mean ± SD. n=3 independent experiments. **p<0.01 *vs* Ca. VSMCs, vascular smooth muscle cells; Ca, calcification medium; Leptin (400), 400 ng/ml; α-SMA, α-smooth muscle actin; DAPI, 4’,6-diamidino-2-phenylindole; GAPDH, glyceraldehyde-3-phosphate dehydrogenase.

To test whether leptin promoted vascular calcification involving in phenotypic switch of VSMCs, marker of VSMCs contractile phenotype was measured. Compared with calcification medium alone, immunofluorescence and Western blot analysis showed that the expression of α-SMA decreased in human VSMCs incubated with leptin and calcification medium. The results were shown in **Figures 3A, B**. The result indicated that leptin promoted phenotypic switch of VSMCs by decreasing the expression of α-SMA.

### Leptin Regulated the Expression of Bone-Related Gene or Protein Markers *via* PI3K/Akt Signaling Pathway in VSMCs *In Vitro*

**Figure 4:**
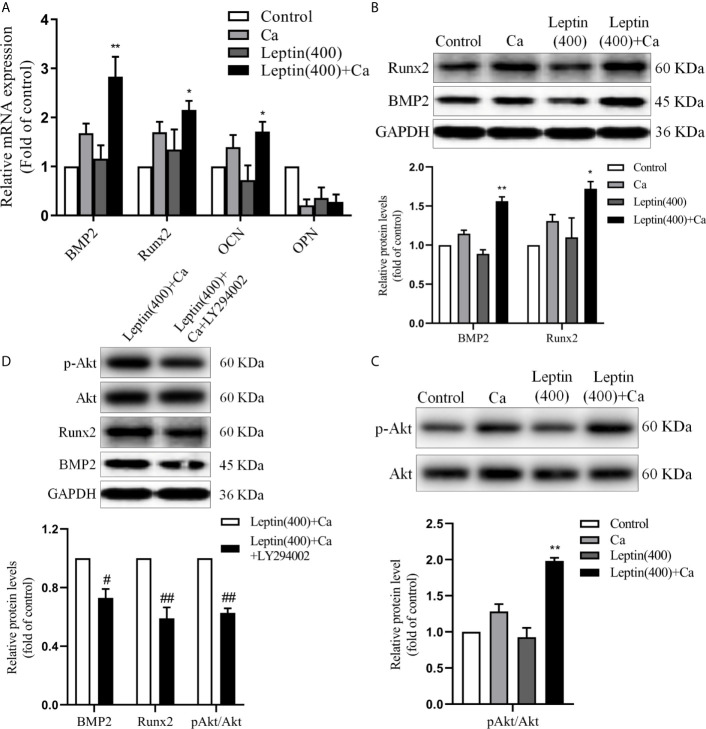
Leptin regulates the expression of bone-related gene markers in VSMCs. **(A)** quantitative RT-PCR assay for expression of BMP2, Runx2, OCN and OPN in VSMCs. Data are mean ± SD. n=6 independent experiments. *p<0.05, **p<0.01 *vs* Ca. **(B)** Western blot assay for expression of BMP2 and Runx2 in VSMCs. Data are mean ± SD. n=3 independent experiments. *p<0.05, **p<0.01 *vs* Ca. **(C)** Western blot assay for Akt and p-Akt in VSMCs. Data are mean ± SD. n=3 independent experiments. **p<0.01 *vs* Ca. **(D)** Western blot assay for expression of Akt, p-Akt, BMP2 and Runx2 in VSMCs. Data are mean ± SD. n=3 independent experiments. ^#^ *p*<0.05, ^##^ *p*<0.01 *vs* Leptin(400)+Ca. VSMCs, vascular smooth muscle cells; Ca, calcification medium; Leptin (400), 400 ng/ml; BMP2, bone morphogenic protein 2; Runx2, runt-related transcription factor 2; OCN, osteocalcin; OPN, osteopontin; Akt, protein kinase B; GAPDH, glyceraldehyde-3-phosphate dehydrogenase.

To explore the possible mechanism of leptin promoting vascular calcification, the markers of bone-related gene or protein were measured. The mRNA level of BMP2, Runx2 and OCN was significantly increased by 68% (*p*<0.01), 27% (*p*<0.05) and 23% (*p*<0.05) in VSMCs incubated with leptin and calcification medium versus calcification medium alone. And the mRNA level of OPN showed no significance in leptin with calcification medium versus calcification medium alone (**Figure 4A**). As compared with calcification medium alone, treatment with leptin and calcification medium increased the protein level of BMP2 and Runx2 (**Figure 4B**). Moreover, the phosphorylation of Akt was upregulated in VSMCs treated with leptin and calcification medium than calcification medium alone (**Figure 4C**).

To examine whether PI3K/Akt signaling pathway involved in the regulation of BMP2 and Runx2, LY294002, the specific inhibitor of PI3K/Akt, was used in further study. As shown in **Figure 4D**, LY294002 decreased the protein expression of Runx2 and BMP2 induced by leptin. These results demonstrated that leptin aggravated vascular calcification by increasing expression of bone related gene or protein *via* PI3K/Akt signaling pathway.

## Discussion

Vascular calcification is a characteristic change of diabetic vascular disease, including intimal and media calcification. Previous studies showed VC is the main independent risk factor for morbidity and mortality of cardiovascular diseases with diabetes (29, 30). Hyperglycemia and increased glycation end products, hyperinsulinemia, oxidative stress, lipid metabolism disturbance, inflammation, apoptosis and abnormal expression of bone regulatory protein are the main factors of vascular calcification in T2DM, which are closely related to the occurrence and development of vascular calcification (31, 32). In a population-based multi-ethnic cohort (33), Katz et al. demonstrated that both metabolic syndrome and diabetes are independently associated with increased prevalence and severity of calcified atherosclerotic plaque in the thoracic aorta. Consistent with our findings, Chen et al. (34) demonstrated that the calcification of VSMCs is significantly enhanced when co-cultured with adipocytes compared to VSMCs alone. A recent study of 548 community-dwelling men reported high leptin levels associated with greater severity and rapid progression of abdominal aortic calcification, independent of smoking or LDL-C or TG levels (35). Moreover, a high baseline level of leptin was found associated with increasing coronary artery calcium severity (36). In according with our results, Tanna et al. (37) indicated, in a cohort of community dwelling post-menopausal women, adiponectin, leptin and vaspin regulate pathways linked with atherosclerosis, VC and stiffness. Wu et al. (38) found that the serum leptin levels were negatively associated with spine, hip and femur bone mineral density (BMD). In a meta-analysis, leptin was positively associated with BMD in post-menopausal women, although the associations were attenuated after adjusting for body mass index (39). It is expected to provide new ideas for the prevention of cardiovascular complications in T2DM by exploring the pathogenesis and relationship between VC and diabetes. In the present study, we found that patients with T2DM showed higher plasma leptin level and median calcification score in lower-extremity artery than control group. Moreover, the results of correlation analysis showed that plasma leptin level was positively correlated with the severity of calcification in T2DM (R^2^ = 0.2184). Our results suggested that plasma leptin could reflect the severity of vascular calcification of lower extremity. The VC process, analogous to that of bone morphogenesis, can generally be described as a form of progressive arterial stiffness that results in reduced vessel compliance and function, ultimately increasing the risk of limb ischemia and lower-extremity amputation [(2, 3, 40, 41). Historically, advancing VC was considered a passive deposition of calcium along the vessel wall. However, VC formation is now considered a complex, actively regulated process, similar to that required for skeletal bone formation.

Osteoblastic differentiation of VSMCs plays an important role in arterial calcification (42). With phenotype switching, VSMCs alternate from contractile (differentiated) phenotype to synthetic (dedifferentiated) phenotype, as well as reduced expression of differentiation marker α-SMA (43). In this study, we detected the expression of α-SMA in calcified VSMCs model using immunofluorescence. The results showed that the fluorescence intensity of α-SMA in leptin with calcification medium was significantly lower than calcification medium alone, Furthermore, compared with calcification medium alone, the protein expression of α-SMA in leptin with calcification medium was decreased. Our results suggested that leptin aggravates the phenotype transition of VSMCs induced by calcification medium, which evidently contributed to the decreased expression of α-SMA.

BMPs are a superfamily of transforming growth factor beta and secretory growth factor, which are reported to have osteogenic actions and play important roles in bone formation. Study reported that vascular calcification is associated with deposition of BMPs in patients with end-stage renal disease, which implied an active cell-mediated process and could be able to intervention (44). The combined use of leptin/leptin receptor and mechanical stress promotes osteogenic differentiation of the posterior longitudinal ligament, accompanied by upregulation of the osteogenic markers osteocalcin and Runx2 (45). Runx2, a key regulator of osteoblast differentiation and bone development, can induce transdifferentiation of VSMCs to an osteochondrocytic phenotype (46). But the exact mechanism of VC in T2DM is still unclear. In this study, we firstly used different concentrations of leptin on VSMCs calcification model. The results showed that leptin could further aggravate calcium deposition of VSMCs. Moreover, leptin with calcification medium could effectively increase the mRNA level of BMP2, Runx2 and OCN versus calcification medium alone in VSMCs. Western blot analysis indicated the protein expression of BMP2 and Runx2 was upregulated by leptin with calcification medium versus calcification medium alone in VSMCs. Consistent with our results, previous studies demonstrated that BMP2 and Runx2 play a crucial role in vascular calcification (46–48). PI3K/Akt signaling pathway was involved in a variety of biological process. In recent years, evidences showed that osteogenesis was regulated by PI3K/Akt signaling pathway (49–51). Our study indicated that inhibition of PI3K/Akt signaling pathway can reverse the expression of osteogenic makers induced by leptin, suggesting that leptin regulates the osteogenic transformation of VSMCs through PI3K/Akt signaling pathway.

The present study demonstrates that VSMCs incubated with leptin and a high calcium environment undergo phenotypic switch that led to some limited osteoblast-like properties. However, this comparison was for *in vitro* studies with calcification induced by increasing both leptin and extracellular calcium level. Ultimately, understanding the differences between VSMCs and osteoblasts may help in developing therapeutic strategies that prevent pathological vascular calcification in T2DM.

There are several limitations in this study. First, we were unable to evaluate the causal relationship between leptin and lower-extremity artery calcification due to the cross-sectional design. Second, CT imaging, which is thought to reflect mainly plaque calcification, cannot precisely distinguish medial from intima calcification.

Our findings suggest that leptin could play a role in the development of lower-extremity artery calcification in T2DM through accelerating calcification of VSMCs *via* PI3K/Akt signaling pathway.

## Data Availability Statement

The raw data supporting the conclusions of this article will be made available by the authors, without undue reservation.

## Ethics Statement

The studies involving human participants were reviewed and approved by The Ethics Committee of Peking University International Hospital. The patients/participants provided their written informed consent to participate in this study.

## Author Contributions

Conception and design: SC and YQ. Analysis and interpretation of data: SC and XM. Investigation: SC, YC, SX, YL, NY, JS, and XM. Writing: SC. All authors contributed to the article and approved the submitted version.

## Funding

This work was supported by the National Natural Science Foundation of China (81700779, 31872790), and by Peking University International Hospital Research Grant (YN2016QN05, YN2017QX02). The Outstanding Clinical Discipline Project of Shanghai Pudong and the program of Shanghai Municipal Health Commission (ZK2019B25).

## Conflict of Interest

The authors declare that the research was conducted in the absence of any commercial or financial relationships that could be construed as a potential conflict of interest.
